# Evidence for Regulation of Hemoglobin Metabolism and Intracellular Ionic Flux by the *Plasmodium falciparum* Chloroquine Resistance Transporter

**DOI:** 10.1038/s41598-018-31715-9

**Published:** 2018-09-11

**Authors:** Andrew H. Lee, Satish K. Dhingra, Ian A. Lewis, Maneesh K. Singh, Amila Siriwardana, Seema Dalal, Kelly Rubiano, Matthias S. Klein, Katelynn S. Baska, Sanjeev Krishna, Michael Klemba, Paul D. Roepe, Manuel Llinás, Celia R. S. Garcia, David A. Fidock

**Affiliations:** 1 Department of Microbiology and Immunology, Columbia University Medical Center, New York, NY, USA; 2 Department of Biological Sciences, University of Calgary, Calgary, Canada; 3 Lewis-Sigler Institute for Integrative Genomics, Princeton University, Princeton, NJ, USA; 4 Department of Parasitology, Institute of Biomedical Sciences, University of São Paulo, São Paulo, Brazil; 5 Department of Physiology and Biophysics, Institute of Biomedical Sciences, University of São Paulo, São Paulo, Brazil; 6 Departments of Chemistry and of Biochemistry and Cellular & Molecular Biology, Georgetown University, Washington, DC, USA; 7 Department of Biochemistry, Virginia Tech, Blacksburg, VA, USA; 8 Institute for Infection and Immunity, St. George’s University Hospital, University of London, London, UK; 9 Departments of Chemistry and of Biochemistry and Molecular Biology and Center for Malaria Research, Pennsylvania State University, State College, PA, USA; 10 Department of Clinical and Toxicological Analyses, School of Pharmaceutical Sciences, University of São Paulo, São Paulo, Brazil; 11 Division of Infectious Diseases, Department of Medicine, Columbia University Medical Center, New York, NY, USA

## Abstract

*Plasmodium falciparum* multidrug resistance constitutes a major obstacle to the global malaria elimination campaign. Specific mutations in the *Plasmodium falciparum* chloroquine resistance transporter (PfCRT) mediate resistance to the 4-aminoquinoline drug chloroquine and impact parasite susceptibility to several partner agents used in current artemisinin-based combination therapies, including amodiaquine. By examining gene-edited parasites, we report that the ability of the wide-spread Dd2 PfCRT isoform to mediate chloroquine and amodiaquine resistance is substantially reduced by the addition of the PfCRT L272F mutation, which arose under blasticidin selection. We also provide evidence that L272F confers a significant fitness cost to asexual blood stage parasites. Studies with amino acid-restricted media identify this mutant as a methionine auxotroph. Metabolomic analysis also reveals an accumulation of short, hemoglobin-derived peptides in the Dd2 + L272F and Dd2 isoforms, compared with parasites expressing wild-type PfCRT. Physiologic studies with the ionophores monensin and nigericin support an impact of PfCRT isoforms on Ca^2+^ release, with substantially reduced Ca^2+^ levels observed in Dd2 + L272F parasites. Our data reveal a central role for PfCRT in regulating hemoglobin catabolism, amino acid availability, and ionic balance in *P. falciparum*, in addition to its role in determining parasite susceptibility to heme-binding 4-aminoquinoline drugs.

## Introduction

Malaria continues to exert a substantial global burden, with an estimated 445,000 deaths in 2016 alone^1^. Global efforts to curtail malaria are challenged by the gain of multidrug resistance in asexual blood stage (ABS) forms of the Apicomplexan parasite *Plasmodium falciparum*, which causes the vast majority of deaths. A central determinant of parasite susceptibility to multiple antimalarials is the *Plasmodium falciparum* chloroquine resistance transporter (PfCRT), a member of the Drug/Metabolite Transporter superfamily that localizes to the digestive vacuole (DV) membrane of intra-erythrocytic ABS parasites^2–4^. Mutant PfCRT is the key determinant of resistance to the former first-line drug chloroquine (CQ), a 4-aminoquinoline that accumulates to micromolar or higher concentrations in the acidic DV wherein its diprotonated form binds to reactive heme and inhibits its detoxification^5–8^.

*P. falciparum* CQ resistance manifests in cultured ABS parasites as a ~5 to 10–fold increase in the IC_50_ value (i.e., the half-maximal inhibitory concentration) of CQ-resistant (CQR) lines relative to CQ-sensitive (CQS) lines^2,9^. Evidence suggests that CQR isoforms of PfCRT mediate the efflux of CQ out of the DV, down the drug’s electrochemical gradient, via a pH-dependent process^10–17^.

Relying on a high electrical membrane-potential difference between the acidic DV (pH 5.2–5.5) and the pH-neutral parasite cytoplasm, CQR isoforms efflux CQ out of the DV, away from this drug’s site of action^10,15–22^. The underlying native function of PfCRT remains unclear, with studies suggesting that PfCRT might transport glutathione, Cl^−^ ions, H^+^ ions, or a range of cationic substrates including hemoglobin (Hb)-derived amino acids or peptides^10,19,23–27^. A recent study using PfCRT-expressing *Xenopus laevis* oocytes also provided evidence that iron may be a substrate of PfCRT^28^. Transport studies also suggest that PfCRT mutations can enable this electrochemical potential-driven transporter to mediate the facilitated diffusion of protonated CQ, which itself might also bind to and inhibit PfCRT^10,12,15,21,29^.

CQ resistance is characterized by its partial sensitization by verapamil (VP), an L-type Ca^2+^ channel blocker^7,30^. Studies with *Xenopus* oocytes have suggested that VP is a partial mixed-type inhibitor of CQR PfCRT-mediated CQ transport^29^. In this model, PfCRT harbors a substrate-recognition cavity with various overlapping substrate binding sites. For instance, it has been suggested that CQ-bound PfCRT predominantly blocks VP, but occasionally permits CQ-VP symport^29^. VP-driven CQ sensitization, or VP reversibility, can be modulated by the PfCRT mutation N326D^31^. This mutation forms part of the minimal set of four mutations (along with K76T, A220S and I356L) that are present in the Ecu1110 haplotype and confer low-level CQ resistance, with a fifth C72S mutation augmenting the degree of resistance^31^.

PfCRT mutations can also decrease or increase parasite susceptibility to several other drugs. These include various partner agents employed in globally adopted artemisinin-based combination therapies, notably piperaquine, amodiaquine (ADQ), lumefantrine, and mefloquine^5,11,32–36^. Consistent with its complex patterns of evolution in multiple geographic regions subject to varied drug selection pressures, there are currently 57 known PfCRT isoforms worldwide, including four observed recently in piperaquine-resistant parasites from Cambodia that have recently been shown to confer piperaquine resistance^17,34,36–38^.

Previously, we reported a novel PfCRT mutation at position 272 (L272F) that produces large, translucent DVs in both the CQS 3D7 line that expresses wild-type PfCRT and the CQR Dd2 line that expresses a variant PfCRT isoform containing 8 point mutations (Supplementary Fig. S1a)^39^. This mutation was observed in 3D7 parasites that had been transfected with a plasmid encoding the blasticidin S-deaminase selectable marker and pressured with blasticidin (a commercial fungicide that inhibits protein translation). This mutation is not present in any of the 2,512 Asian or African *P. falciparum* genomes deposited by the MalariaGEN consortium (www.malariagen.net/pf3k) or in any cultured laboratory lines that we know of, other than the blasticidin-selected 3D7^*L272F*^ or *pfcrt*-edited Dd2^*Dd2 L272F*^ lines^39–41^. L272 is predicted to be situated at the start of a long loop between transmembrane helices 7 and 8, adjacent to the Dd2 Q271E mutation that has been implicated in CQ transport and resistance^16^. Using zinc-finger nucleases, we earlier introduced the L272F mutation into a CQR Dd2 line and observed a substantially enlarged DV (as visualized on thin blood smears; exemplified in Supplementary Fig. S1b)^39^. The L272F mutation also increased parasite susceptibility to the 4-aminoquinoline compounds CQ, monodesethyl-CQ (md-CQ), and monodesethyl-ADQ (md-ADQ). The PfCRT variant Dd2 + L272F, heterologously expressed in *Xenopus* oocytes, exhibits significantly impaired CQ transport^39^.

Given our initial findings, we sought to further investigate the effect of the PfCRT L272F mutation. Previously, PfCRT isoforms have been associated with altered Hb catabolism in the DV^26,28^. Hb is present at low millimolar concentrations in the RBC cytoplasm and is imported into the DV via cytostomes or endocytic vesicles^42,43^. In the DV, Hb catabolism employs aspartic and cysteine proteases that generate oligopeptides^44^ as well as free Fe^2+^-heme (which is biomineralized into chemically inert hemozoin^8,45–47^). Oligopeptides are degraded by the Cl^−^dependent exopeptidase dipeptidyl aminopeptidase 1 (PfDPAP1) and the Zn^2+^ and/or Mn^2+^-dependent peptidases falcilysin, aminopeptidase P (PfAPP), and aminopeptidase N (PfA-M1). These peptidases generate short peptides and amino acids, which can be transported out of the DV^48–52^. During one 48-hour intra-erythrocytic development cycle, parasites degrade ~75% of the Hb content of infected RBCs (iRBCs)^53–55^, while only metabolizing ~16% as a nutrient source^54^ and releasing the majority of the remainder into host plasma^56^.

Inhibition of Hb catabolism generates two distinct enlarged DV phenotypes. Parasites treated with the protease inhibitors N-acetyl-leucinyl-leucinyl-norleucinal (ALLN) or *trans*-Epoxysuccinyl-L-leucylamido(4-guanidino)butane (E64) produce dark, enlarged DVs in Giemsa-stained thin blood smears^57–59^, indicating excess globin accumulation. ALLN and E64 are predicted to target parasite proteases including calpains and cysteine proteases, respectively. In contrast, inhibition of aminopeptidases such as PfA-M1 produces translucent, enlarged DVs, indicating accumulation of short oligopeptides^60^. Mutations in PfCRT or inhibition by drugs can also increase DV volume. For instance, the CQR Dd2 isoform produces larger DV volumes compared to the CQS isoform^18^.

Given that the L272F mutation produces translucent, distended DVs^39^, we hypothesized that this mutation may impact Hb processing in the DV. We engineered Dd2 parasites to express the Dd2 + L272F variant and, by peptidomic analysis, observed an elevated peptide profile also seen with the Dd2 isoform. Our data also revealed impaired efflux of free Ca^2+^ across the DV membrane in L272F parasites stimulated by ionophores. Comparison of isogenic lines expressing Dd2 PfCRT or the Dd2 + L272F variant indicated that the L272F mutation confers a fitness cost, causes methionine auxotrophy, and modulates parasite susceptibility to several antimalarial drugs. Our findings support the hypothesis that PfCRT is involved in regulating parasite access to globin-derived peptides that are necessary for *de novo* protein synthesis, and balancing ionic homeostasis in the DV that is a key factor in maintaining the catalytic efficiency of Hb peptidases.

## Results

### The PfCRT L272F mutation reverses 4-aminoquinoline drug resistance independently of verapamil (VP)

To define the impact of the L272F mutation on both CQ susceptibility and drug potentiation by VP, we examined four *P. falciparum* lines: Dd2 (a CQR parental control), Dd2^*Dd2*^ (a recombinant isogenic control), Dd2^*Dd2 L272F*^, and GC03 (a CQS control). Parasite lines Dd2^*Dd2*^ and Dd2^*Dd2 L272F*^ were generated previously using zinc-finger nuclease-mediated genome editing^39,61^. Each line was exposed to the 4-aminoquinoline compounds CQ, md-CQ, or md-ADQ tested across a range of concentrations ±0.8 μM VP (see Methods).

**Figure 1:**
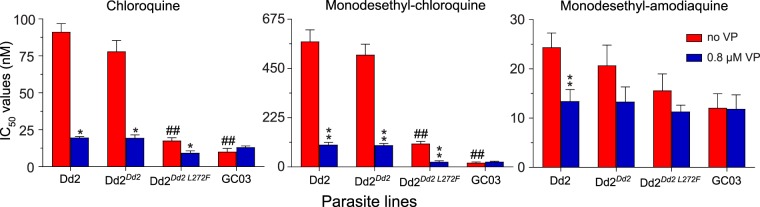
*In vitro* susceptibility of *pfcrt*-modified lines to 4-aminoquinoline drugs tested ± the resistance reversal agent VP. Graphs show mean ± SEM IC_50_ values for Dd2, Dd2^*Dd2*^, Dd2^*Dd2 L272F*^, and GC03 in response to CQ, md-CQ, and md-ADQ ± 0.8 μM VP. IC_50_ values without VP were previously published^39^. VP results were obtained in parallel in the same sets of assays. Data were generated in duplicate on 4 to 6 independent occasions (see Supplementary Table S1). Statistical comparisons were made using two-tailed Mann-Whitney *U* tests. *p < 0.05, **p < 0.01, relative to the no-VP IC_50_ value in the same line. ^##^*p* < 0.01, relative to Dd2^*Dd2*^.

Our data show that in the Dd2^*Dd2*^ background the addition of L272F resulted in a 4.4–fold reduction in the mean CQ IC_50_ (i.e., when comparing Dd2^*Dd2*^ and Dd2^*Dd2 L272F*^; *p* < 0.01, Mann-Whitney *U* test; Fig. 1; Supplementary Table S1). Comparisons between Dd2^*Dd2 L272F*^ and the CQR Dd2^*Dd2*^ and the CQS GC03 lines showed that the Dd2 + L272F isoform ablated most of the resistance phenotype but did not fully confer CQ sensitivity. In the Dd2^*Dd2*^ line, VP reversibility was observed as a 4.0–fold decrease in CQ + VP IC_50_ values compared to CQ only. For Dd2^*Dd2 L272F*^, the reversibility was less pronounced, equating to a 1.9–fold decrease. In the presence of VP, the Dd2^*Dd2 L272F*^ line showed a CQ IC_50_ value that was close to that of the CQS GC03 line (Fig. 1; Supplementary Table S1). md-CQ assays yielded IC_50_ values in Dd2^*Dd2 L272F*^ that were 4.8–fold lower as compared with Dd2^*Dd2*^, yet remained 5.5–fold above those of GC03. These data with the md-CQ metabolite show a much higher level of resistance imparted by the Dd2 allele as compared with its effect on the parent compound CQ, consistent with the idea that md-CQ might be the more important selective agent^31^. md-CQ results also showed some degree of VP reversibility with the L272F mutant. We consider the Dd2^*Dd2 L272F*^ line to be CQ-tolerant, in accordance with the earlier definition of tolerant parasites having IC_50_ values that for CQ are only nominally higher than CQS lines yet for md-CQ are intermediate between CQS and CQR lines^62^. For md-ADQ (the active metabolite of ADQ), drug assays yielded slightly lower IC_50_ values in the Dd2^*Dd2 L272F*^ line, accompanied by a slight reduction in the degree of VP reversibility, compared to the parental Dd2^*Dd2*^ line. These differences, however, were not statistically significant (Fig. 1; Supplementary Table S1).

Parallel studies with lumefantrine, mefloquine, and pyronaridine found no significant differences in IC_50_ values between Dd2^*Dd2 L272F*^ and Dd2^*Dd2*^. These findings are consistent with PfCRT not being a major modulator of *P. falciparum* response to these first-line partner drugs^4^ (Supplementary Table S1).

### The L272F mutation results in enlarged DV volumes and a growth defect

**Figure 2:**
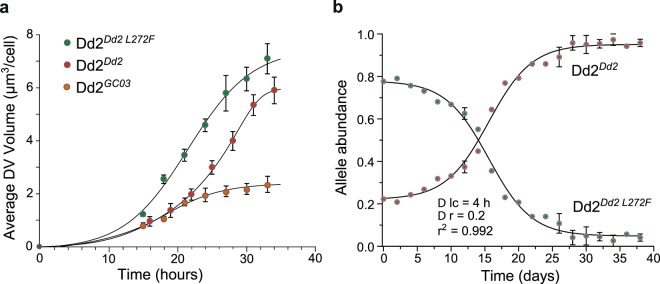
The PfCRT L272F mutation causes an increase in the DV volume and exerts a fitness cost. (**a**) Average DV volumes of the *pfcrt*-modified Dd2^*Dd2 L272F*^ (green), Dd2^*Dd2*^ (red) and Dd2^*GC03*^ (dark orange) lines over a 36-hour time course. DV volumes are represented as the means ± SEM of 15 independent experiments. (**b**) Changes in allele frequencies over time, as derived from head-to-head *in vitro* competition assays between Dd2^*Dd2*^ and Dd2^*Dd2 L272F*^. Dd2^*Dd2*^ and Dd2^*Dd2 L272F*^ were seeded at a 1:4 ratio and monitored every 2 days for 40 days (i.e. 20 generations). After 4 generations, cultures were split into two replicates. Observed allele frequencies (shown as means ± SEM beginning on the 5^th^ generation, i.e., day 10) were fitted using a computer model of the *Plasmodium* life cycle (solid lines, yielding a very close correlation with r^2^ = 0.992). Results indicated a 20 ± 1% fitness difference and 4.0 ± 0.2 hour increase in the cell cycle duration in the Dd2^*Dd2 L272F*^ line. D lc h, difference in the intra-erythrocytic life cycle in hours; D r, difference in relative fitness.

Previously we presented evidence to suggest that the L272F mutation increases the surface area of DV compared to a Dd2^*Dd2*^ control, as estimated by light microscopy^39^. Accurate quantification, however, was not possible with these earlier two-dimensional surface area estimates. Using spinning disk confocal microscopy^18^, we have now measured DV volume changes over time for three lines: Dd2^*Dd2*^, Dd2^*Dd2 L272F*^, and the previously generated wild-type GC03 *pfcrt*-encoding recombinant CQS line Dd2^*GC03*^ ^61^. Volumes were measured for up to 36 hours during the intra-erythrocytic life cycle prior to schizogony (Fig. 2a; Supplementary Table S2). Results revealed a substantial increase in DV volume over time in both the Dd2^*Dd2 L272F*^ and Dd2^*Dd2*^ parasite lines, with the largest increase observed in Dd2^*Dd2 L272F*^. We observed ~1.3–1.9–fold increase in the DV volume in the Dd2^*Dd2 L272F*^ parasite line compared to the Dd2^*Dd2*^ control. This increase in the DV volume attained significance between 18–27 hours post-invasion (*p* < 0.001, Mann-Whitney *U* tests). Increases over time were more modest in the Dd2^*GC03*^ line, particularly in the latter stage of trophozoite development, with a calculated volume at ~33 hours post-invasion that was 3–fold less than Dd2^*Dd2 L272F*^ (Fig. 2a; Supplementary Table S2).

Prior studies have established that sequence changes in PfCRT can alter growth rates *in vitro*, a proxy of ABS parasite fitness^26,31,32,34^. For instance, parasites expressing the mutant *pfcrt* Dd2 allele were found to be less fit than the isogenic line expressing the wild-type (GC03) allele. This fitness cost was exacerbated when Dd2 was propagated in media lacking all amino acids except isoleucine, which is absent in Hb and therefore needs to be supplied exogenously^26^. In this minimal media, parasites must derive all amino acids (except isoleucine) from Hb. These earlier findings suggested that PfCRT mutations can impair amino acid availability in ABS parasites^26,34^.

To test for altered growth rates, we seeded Dd2^*Dd2 L272F*^ and Dd2^*Dd2*^ in a 4:1 ratio in an *in vitro* head-to-head competition and monitored *pfcrt* allele frequency every 48 hours for 40 days (~20 generations). We chose an increased abundance of the Dd2^*Dd2 L272F*^ mutant line at the outset of the competition assay to compensate for its observed slower growth rate compared with Dd2^*Dd2*^. Results shown in Fig. 2b showed a substantial fitness cost for Dd2^*Dd2 L272F*^, suggesting that the L272F mutation has a major adverse impact on native PfCRT function. We then employed a published customized computer model of the *Plasmodium* life cycle^26^ that showed an excellent fit with our competition data (r^2^ = 0.992). This model predicted a 4.0 ± 0.2 hour delay in the asexual cell cycle length and a 20 ± 1% relative fitness difference between Dd2^*Dd2 L272F*^ and Dd2^*Dd2*^ (Fig. 2b).

### The L272F mutation confers methionine auxotrophy

**Figure 3:**
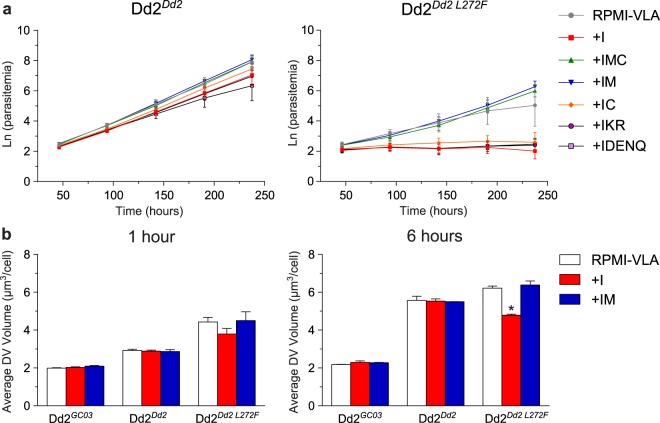
Parasites harboring the PfCRT L272F mutation require methionine for growth. (**a**) Growth rates of Dd2^*Dd2*^ (left panel) and Dd2^*Dd2 L272F*^ (right panel) in amino acid-rich media (RPMI-VLA) and minimal media (lacking all amino acids save for supplementation with the ones denoted by single letter codes, e.g. IM: isoleucine plus methionine). Note that for Cysteine (C), this was supplemented as the oxidized dimer cystine that is presumably reduced in the host RBC. Unlike Dd2^*Dd2*^, Dd2^*Dd2 L272F*^ cannot grow in medium containing only isoleucine, but can be rescued with the addition of methionine (IM and IMC). Cumulative parasitemia expansions, calculated as Ln-transformed parasitemias, were calculated from three independent experiments for each line and media condition. Data are presented as the natural log of parasitemia adjusted for dilution factor over 240 hours. Student *t* tests performed at the last time point showed significant differences only for the Dd2^*Dd2 L272F*^ line cultured in +I versus +IM or +IC versus +IMC. For this parasite line, no significant differences were observed between RPMI-VLA and any other media. All comparisons for different media were non-significant with Dd2^*Dd2*^ parasites. (**b**) Average DV volumes (represented as μm^3^/cell) of Dd2^*GC03*^, Dd2^*Dd2*^ and Dd2^*Dd2 L272F*^ lines post amino acid-starvation for either 1 hour (left panel) or 6 hours (right panel). DV volumes are represented as mean ± SEM calculated from 30 independent experiments. Statistical comparisons used Mann-Whitney *U* tests, which for each line compared volumes in amino-acid depleted media vs. those in rich RPMI-VLA media. *p < 0.05.

Cultured *P. falciparum* parasites are known to rely on Hb catabolism as a source of all amino acids required for growth, except isoleucine that is absent from Hb^63^. To determine whether the L272F mutation impairs the ability of parasites to secure essential amino acids from digested Hb, we profiled the amino acid requirements in the L272F mutant. We focused our analysis on amino acids that appear to not be effluxed from the parasite in substantial quantities, namely Methionine (M), Cysteine (C; supplemented as the oxidized dimer Cystine that is presumably reduced in the host RBC), Aspartate (D), Asparagine (N), Glutamate (E), Glutamine (Q), Lysine (K) and Arginine (R)^56^. Growth conditions were profiled in media containing amino acid-rich media (RPMI-VLA), which has all amino acids other than valine, leucine, and alanine and which allows unhindered parasite growth. Growth in that media was compared with growth in minimal (amino-acid deficient) media that contained only amino acids added separately or as restricted combinations (minimal media +I, +IMC, +IM, +IC, +IKR, or +IDENQ)^56^. Dd2^*Dd2*^ showed good growth in all media conditions (Fig. 3a). A very different outcome was observed with Dd2^*Dd2 L272F*^, which was unable to replicate in minimal media (+I) that had only isoleucine supplementation, or in +IC, +IKR or +IDENQ media. Strikingly, Dd2^*Dd2 L272F*^ growth returned to RPMI-VLA levels upon supplementation of minimal media with +IM or +IMC. Growth was significantly improved in +IM media compared with +I, or in +IMC media compared with +IC (Student *t* test, *p* < 0.05 for both comparisons at the last time point of 238 hours). These findings provide evidence that the L272F mutation causes the Dd2 parasite to become a methionine auxotroph. Parasitemias remained lower overall with Dd2^*Dd2 L272F*^ compared with *Dd2*^*Dd2*^, which is consistent with the reduced growth rate imposed by the L272F mutation (Fig. 2b).

To determine the effect of amino acid starvation on DV volume, we calculated the volume of dextran-conjugated Orange Green-loaded DVs for Dd2^*Dd2*^, Dd2^*Dd2 L272F*^, and Dd2^*GC03*^ (Fig. 3b). Synchronized early trophozoites were propagated for 1 or 6 hours in RPMI-VLA, +I, or +IM media and visualized. Dd2^*Dd2*^ and Dd2^*GC03*^ DV volumes were unaffected by minimal media conditions +I or +IM, compared with the amino acid-rich media RPMI-VLA. The average DV volumes across all media conditions for Dd2^*Dd2*^ were 1.4– and 2.5–fold higher than the average Dd2^*GC03*^ DV volumes at 1 and 6 hours, respectively, in accordance with our data in Fig. 2a. Relative to both control line DV volumes, Dd2^*Dd2 L272F*^ DV volumes were increased in RPMI-VLA and +IM conditions, but not +I. After 6 hours of trophozoite growth in +I media, the Dd2^*Dd2 L272F*^ DV volume was 23% less than the DV volume in RPMI-VLA. Notably, the DV volume was restored to levels observed in RPMI-VLA media when methionine was added to the +I media. This finding suggests that the L272F mutant might be impaired in its methionine transport.

These data provide compelling evidence that PfCRT is an important factor in the availability of amino acids, particularly methionine, for cellular processes and growth. However, these data do not mandate that PfCRT is an amino acid or methionine transporter. Methionine is a low abundance amino acid in Hb (1.7%), and some lines have been found to require methionine in addition to isoleucine for efficient *in vitro* culture propagation^63^. Therefore, methionine auxotrophy might be a consequence of the L272F mutation’s impact on PfCRT, as opposed to the alternative inference that methionine might be a physiologic substrate. These data nonetheless highlight the close relationship between PfCRT and Hb catabolism.

### The L272F mutant accumulates short, Hb-derived peptides

**Figure 4:**
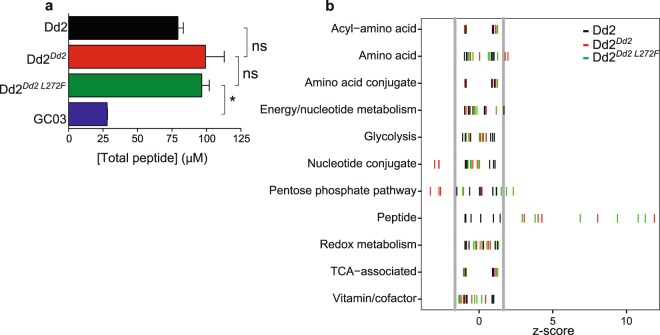
The PfCRT L272F mutant accumulates peptides to the same degree as Dd2^*Dd2*^. (**a**) Total concentrations per parasite of short, Hb-derived peptides for Dd2 (black), Dd2^*Dd2*^ (red), Dd2^*Dd2 L272F*^ (green), and the CQS line GC03 (blue) as measured by LC-MS. The increase in peptide levels in the Dd2 lines compare to GC03 was irrespective of the source of Hb subunit (α or β; data not shown). Data from all lines were generated at the same time. Dd2^*Dd2 L272F*^ had significantly higher total peptide concentrations than GC03 (*p < 0.05; two-tailed Student *t* test), but did not differ statistically from Dd2 or Dd2Dd2(ns). (**b**) Standard scores (z-scores) of metabolites measured by LC-MS for Dd2 (black), Dd2^*Dd2*^ (red), and Dd2^*Dd2 L272F*^ (green). Each bar represents the number of standard deviations for each metabolite such that z = (x − μ)/σ, where x = mean signal of a metabolite (e.g. PEEK) for 3 independent assays, μ = mean signal of a compound class (e.g. “peptide”), and σ = the standard deviation for the same compound class in Dd2. This method of analysis allowed for direct comparisons across distinct metabolite classes^34^. Vertical grey lines represent lower (5%) and upper (95%) boundaries for the normal distribution, as defined for Dd2 parasites.

To further investigate the link between the L272F mutation and Hb catabolism, we examined the accumulation of peptides in the L272F mutant. For this, we analyzed the metabolites of magnetically purified Dd2, Dd2^*Dd2*^, Dd2^*Dd2 L272F*^, and GC03 parasites harvested at an average of ~38 hours post-invasion (Fig. 4; Supplementary Figs S2, S3). These studies used high-resolution mass spectrometry-based profiling of synchronous trophozoite-stage cultures (24 hours post-invasion), as previously described^26^.

Consistent with an earlier report that observed a peptide accumulation phenotype in CQR lines^26^, we observed a 50 to 70 μM increase in Hb-derived total peptide concentration in the CQR lines Dd2 and Dd2^*Dd2*^, relative to the CQS line GC03. This earlier study had postulated that a feature of CQR parasite lines expressing mutant PfCRT isoforms was an increase in Hb peptide levels. Our findings here refine this interpretation by revealing that the CQ-tolerant Dd2^*Dd2 L272F*^ line also exhibited a total peptide concentration (Fig. 4a) and mean Z-scores for peptide species (Fig. 4b) that were similar to the recombinant CQR control Dd2^*Dd2*^. Compared with GC03, the increase in total peptide concentrations in Dd2^*Dd2 L272F*^ parasites was significant (*p* < 0.05). Both Dd2^*Dd2*^ and Dd2^*Dd2 L272F*^ parasites appeared to have slightly increased peptide concentrations relative to Dd2, although this did not reach statistical significance.

Total peptide concentrations remain unchanged between Dd2^*Dd2*^ and Dd2^*Dd2 L272F*^, with most individual peptide profiles being similarly elevated with respect to GC03 (Supplementary Figs S2, S3). Only PEEK was found to be significantly elevated in Dd2^*Dd2 L272F*^ compared with Dd2^*Dd2*^. Our earlier study had observed that levels of PEEK were significantly increased in parasites expressing the Dd2 isoform compared to the wild-type isoform^26^, suggesting that PfCRT mutations play an important role in dictating levels of this oligopeptide in parasites.

### The L272F mutation restricts free Ca^2+^ efflux upon ionophore stimulation

The excess accumulation of Hb-derived peptides in our variant PfCRT lines suggested impaired processing by terminal peptidases, which could be attributable either to decreased efflux of peptides from the DV to the cytosol whereupon they could be digested by cytosolic peptidases, or a change in the ionic homeostasis of the DV itself that impacts the kinetics of DV-resident peptidases. Of note, prior studies have associated the concentration of several ions (including Zn^2+^, Mn^2+^, Mg^2+^, Co^2+^, and Cl^−^ that have so far been studied) with activity of the terminal peptidases PfAPP, PfA-M1, PfDPAP1, and falcilysin^48,51,52^. To explore whether PfCRT isoforms might impact ionic homeostasis, we examined the effect of the L272F mutant on free Ca^2+^ in the parasite. We focused on Ca^2+^ also because of its known involvement in cytoplasmic protease activity, host-cell invasion, and intra-erythrocytic development, and the existence of robust methods of Ca^2+^ quantification^64–67^. Furthermore, the DV is believed to be a secondary source of intracellular Ca^2+^, with the primary store located in the endoplasmic reticulum^65,68–70^. Free Ca^2+^ release from the DV was also reported in a recent study to be associated with the CQ susceptibility status of parasite lines^71^. We hypothesized that if PfCRT could modulate cation stores, possibly by impacting their transport across the DV, its haplotypes might affect the levels of free Ca^2+^ in the cytoplasm upon ionophore stimulation^64,72^.

Ca^2+^ release from the DV is triggered by the redistribution of the ionic balance and disruption of membrane potentials caused by ionophore treatment^64,72^. To stimulate a free Ca^2+^ response from the DV, we treated parasites with the Na^+^/H^+^ ionophore monensin or the K^+^/H^+^ ionophore nigericin. Monensin and nigericin are lysosomotropic carboxylic ionophores that reversibly form complexes with monovalent ions and act directly as Na^+^/H^+^ and K^+^/H^+^ exchangers to disrupt Na^+^ and K^+^ electrochemical gradients, respectively^64^. We also tested CQ, which has been reported previously to act as a Zn^2+^ ionophore in human lysosomes^71–73^, and to disrupt calcium homeostasis within the DV^74–77^. We monitored changes in free Ca^2+^ levels in the cytoplasm using the fluorescent Ca^2+^ indicator Fluo-4 AM, combined with the organic anion transport blocker probenecid that prevents accumulation of this Ca^2+^-binding dye in the DV^72^.

**Figure 5:**
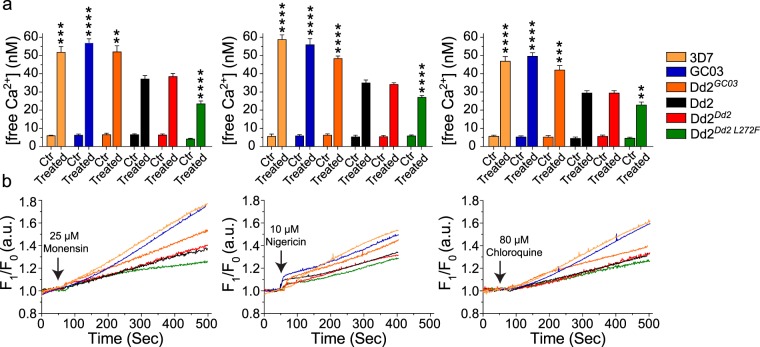
PfCRT L272F mutant DV elicits less calcium efflux upon ionophore treatment. The efflux of free Ca^2+^ from the DV into the cytoplasm was measured in ionophore-treated parasites using the fluorescent dye Fluo-4 AM for 3D7 (light orange), GC03 (blue), Dd2^*GC03*^ (dark orange), Dd2 (black), Dd2^*Dd2*^ (red), Dd2^*Dd2 L272F*^ (green). Each line was independently treated with monensin, nigericin, or CQ. (**a**) Mean free Ca^2+^ concentrations for ionophore-treated or mock-treated (Ctr) parasites. Free calcium measurements were done from the time of monensin, nigericin and chloroquine addition (50–55 sec) until thapsigargin addition at approximately 500 seconds. Levels of free Ca^2+^ flux are shown as means ± SEM, calculated from nine independent experiments. All three CQS lines expressing wild-type *pfcrt*, namely 3D7, GC03 and Dd2^*GC03*^, effluxed significantly higher levels of free Ca^2+^ than the CQR line Dd2^*Dd2*^, whose free Ca^2+^ levels were very similar to non-recombinant Dd2. The Dd2 + L272F variant, which was largely sensitized to CQ (Fig. 1), showed the lowest rate of Ca^2+^ efflux. **p < 0.01, ***p < 0.001, ****p < 0.0001; two-tailed Mann-Whitney *U* test relative to Dd2^*Dd2*^. (**b**) Representative fluorescence intensity (F_1_/F_0_) over a time course where F_1_ was the Fluo-4 AM signal intensity of ionophore-treated parasites; and F_0_, the signal intensity for control treatments.

Studies were conducted with six parasite lines: Dd2, Dd2^*Dd2*^, Dd2^*Dd2 L272F*^, and the three CQS lines GC03, 3D7, and Dd2^*GC03*^ (Fig. 5). 3D7 served as an additional CQS control while Dd2^*GC03*^ served as an isogenic background control to distinguish between the effects of various PfCRT haplotypes expressed on a common genetic background (Dd2). A possible association between PfCRT haplotype and free Ca^2+^ release from the DV was earlier reported^71^, but those experiments did not control for line-dependent differences.

In our assays, we observed that upon stimulation with monensin, nigericin, or CQ, free Ca^2+^ release from the DV in Dd2^*Dd2*^ was reduced ~1.4–fold (for each treatment agent) when compared with the CQS isogenic control Dd2^*GC03*^ (*p* < 0.01; Fig. 5a). Relative to Dd2^*Dd2*^, the L272F mutant further reduced free Ca^2+^ release by 1.6–fold (*p* < 0.0001), 1.2–fold (*p* < 0.0001), and 1.3–fold (*p* < 0.01), upon treatment with monensin, nigericin and CQ, respectively. Indeed, the L272F mutant showed the lowest free Ca^2+^ levels of all, despite the fact that this line has lost most of its CQ resistance. Interestingly, the expression of the wild-type *pfcrt* allele in Dd2 resulted in elevated free Ca^2+^ concentrations that matched those observed with the other CQS parasites 3D7 and GC03. Kinetics of Ca^2+^ release in a single representative experiment are depicted in Fig. 5b. In further studies, the decrease in Ca^2+^ levels in Dd2^*Dd2 L272F*^ parasites observed with ionophore treatment was reversed by adding thapsigargin, a SERCA-pump inhibitor that impacts Ca^2+^ release from endoplasmic reticulum stores (Fig. 5a; Supplementary Fig. S4). Our data therefore suggest an important role for PfCRT mutations in regulating DV membrane permeability to Ca^2+^.

## Discussion

PfCRT is an essential factor for *P. falciparum* intra-erythrocytic development and a key modulator of parasite susceptibility to multiple antimalarials, including being a core mediator of resistance to CQ and ADQ^4,9^. Here, we probed the functional and drug resistance properties of PfCRT by exploring the L272F mutation, which we have previously shown to enlarge the DV and to inhibit the transport of 4-aminoquinoline drugs^39^. When introduced into a Dd2 PfCRT isoform that normally imparts CQ resistance, L272F nearly fully sensitized parasites to CQ, its metabolite md-CQ, and the related drug ADQ, while appearing to retain most of the resistance reversal properties of VP. In the poly-specific substrate-recognition cavity model of PfCRT^78^, the bulky phenylalanine at residue 272 would be predicted to impair quinoline access to PfCRT, but perhaps not interfere with VP sensitization. In this scenario, CQ, md-CQ, or md-ADQ molecules that manage to bypass the L272F mutation such that they could be effluxed by the variant transporter might be inhibited by the presence of VP occupying an overlapping binding site. Our data evoke a mechanism by which the L272F mutation can mostly sensitize CQR parasites to 4-aminoquinoline drugs independent of VP.

We observed an up to 3.5–fold increase in DV volume over 36 hours of intra-erythrocytic growth upon addition of the L272F mutation to the Dd2 isoform. This striking increase was accompanied by a profound growth defect, measured as a 20.0 ± 1.0% diminished growth rate relative to Dd2 and a 4.0 ± 0.2 hour extension in the intra-erythrocytic developmental cycle. Growth in amino acid-depleted medium was virtually eliminated unless exogenous methionine, a low abundance amino acid, was reintroduced. Furthermore, growth in the absence of methionine limited the increase in DV volume of the L272F mutant compared to amino acid-rich media. Our data also document a PfCRT L272F-dependent pattern of accumulation of Hb-derived small peptides and suggest a role of PfCRT isoforms in the regulation of Ca^2+^ levels in the DV.

Our observed pattern of peptide accumulation in Dd2, Dd2^*Dd2*^, and Dd2^*Dd2 L272F*^ parasites supports a model where PfCRT mutations lead to the attenuation of terminal peptidase-mediated steps in Hb catabolism. The three metallopeptidases falcilysin, PfAPP, and PfA-M1, as well as the Cl^−^ ion-dependent PfDPAP1, are involved in this process^49–51,79^. Inhibition of PfA-M1 has been shown to yield enlarged, translucent DVs and an accumulation of Hb-derived peptides that are slightly longer than those in Dd2^*Dd2 L272F*^ (e.g. LTPEEKSAVTALW, compared to PEEK)^60^. In our Dd2^*Dd2 L272F*^ mutant, we also observed some modulation of the profile of Hb-derived peptides relative to the recombinant control Dd2^*Dd2*^ (Supplementary Figs S2, S3). For instance, PEEK, but not PE, appeared preferentially enriched in Dd2^*Dd2 L272F*^ parasites, suggesting that a specific peptidase that acts on PEEK might be less active in the L272F mutant. PfDPAP1 is a plausible candidate for this activity, given its likely role in the catabolism of vacuolar oligopeptides and its ability to accept a proline residue in the P2 position (substrate residues are identified using the Schechter and Berger nomenclature)^79,80^. The PfDPAP1-catalyzed hydrolysis of PEEK, however, may be rather slow, as a P1-glutamate has been shown to greatly reduce catalytic efficiency^79^. Therefore, the effects of attenuated PfDPAP1 activity may be best observed with substrates with lower hydrolysis rates such as PEEK (Supplementary Fig. S2)^79^. The known P1 and P1’ specificities of PfAPP and PfA-M1 are not consistent with these other exopeptidases having a role in the hydrolysis of PEEK^51,81^.

Our peptidomics data also revealed similar increased overall levels of Hb-derived peptides in Dd2^*Dd2*^ and Dd2^*Dd2 L272F*^, relative to GC03, consistent with a previous report and the hypothesis that mutations in PfCRT impair fitness^26^. PfCRT mutants displayed an overall increase in Hb-derived peptide levels. However, CQ susceptibility or *in vitro* growth rates did not correlate with peptide accumulation, as the Dd2^*Dd2 L272F*^ mutant was both less CQR and less fit than Dd2^*Dd2*^, yet both lines had equivalent peptide levels. Our data therefore suggest that changes in globin catabolism are likely not the predominant driver of the reduced fitness in mutant *pfcrt* lines, evoking instead a convergence of multiple physiological consequences that arise from mutant PfCRT. Given that overall peptide levels seen in the L272F mutants were comparable to Dd2, but the L272F mutants have a larger DV, the intravacuolar peptide concentrations of L272F parasites may actually be lower than those found in Dd2 parasites. In addition to the assays reported herein, mutant PfCRT isoforms have also been observed to impact DV pH and basal levels of free heme, in addition to their contribution to membrane potential-sensitive CQ transport^17,34,82^.

To further explore the physiologic impacts of PfCRT sequence changes, we exploited the ability of the acidic DV to mobilize Ca^2+^ into the cytosol, as previous reports indicated that disrupting the ionic environment of DV releases Ca^2+^ ^72^. Addition of the Na^+^/H^+^ or K^+^/H^+^ ionophores monensin or nigericin, respectively, to Fluo-4AM–loaded trophozoites increased cytosolic Ca^2+^, as did the addition of CQ (at concentrations that alkalize the DV). In our study, we found that the *pfcrt* wild-type CQS lines 3D7 and GC03 released significantly higher Ca^2+^ than the CQR line Dd2 and its recombinant counterpart Dd2^*Dd2*^. Ca^2+^ release was further reduced in the Dd2^*Dd2 L272F*^ line expressing the L272F mutation. This Ca^2+^ mobility was restored in recombinant Dd2 parasites expressing wild-type *pfcrt* (Dd2^*GC03*^). These results lead us to propose that changes in PfCRT are critical for Ca^2+^ mobilization in *Plasmodium*. Our data suggest that along with the endoplasmic reticulum, the DV is fundamental for Ca^2+^ homeostasis in *P. falciparum* ABS parasites, and that disruption of Ca^2+^-dependent signaling pathways could be a promising strategy for the development of new antimalarials.

Together, our study reveals an unexpected relationship between Ca^2+^ release, Hb processing, overall parasite fitness, and the regulation of these parameters by changes in PfCRT. This suggests that PfCRT may be an H^+^-coupled poly-specific cationic nutrient transporter, as previously evidenced in proteoliposome studies^27^. In this model, the PfCRT L272F mutant would be partially inhibited in transporting Ca^2+^, short Hb-derived peptides, 4-aminoquinoline drugs, and VP (Fig. 1). We speculate that this broad substrate specificity is consistent with the polymorphic nature of PfCRT^17^ and its ability to transport multiple drugs^29^.

While our data fit this model, we cannot definitively conclude that PfCRT transports a variety of cationic substrates and acknowledge the possibility that PfCRT may transport other unknown substrates. Given the central role of PfCRT both in parasite development and in resistance to heme-binding antimalarials, there is a pressing need to elucidate its biological functions in the context of parasite physiology and to leverage these insights into identifying novel approaches to combat *P. falciparum* multidrug resistance.

## Methods

### Parasite culturing

Asexual blood stage parasites were maintained in human O^+^ RBCs in RPMI-1640 with 11 mM glucose, supplemented with 2 mM L-glutamine, 25 mM HEPES, 2 g/L sodium bicarbonate, 10 µg/mL gentamycin, 50 µM hypoxanthine and 0.5% (w/v) AlbuMAXII (Thermo Fisher Scientific) under 5%/O_2_/5% CO_2_/90% N_2_, as described^83^. O^+^ RBCs were obtained from Interstate Blood Bank, pooled from anonymous donors without any identifiers. As such, the use of these RBCs is not human subjects research, under the NIH Exemption 4 category. A protocol describing the use of these RBCs for *P. falciparum* research has been approved by the Columbia University Institution Review Board, who classified this work as not being human subjects research. Informed consent does not apply to this study. All methods described herein were carried out in accordance with relevant guidelines and regulations.

### *In vitro* susceptibility assays

*In vitro* IC_50_ values were determined by incubating parasites for 72 hours across a range of concentrations of CQ (0.4 nM to 193.9 nM alone, or 3.8 nM to 1940 nM in the presence of 0.8 μM VP), md-CQ (0.3 nM to 172 nM), md-ADQ (1.9 nM to 998 nM), lumefantrine (0.6 nM to 284 nM), pyronaridine (0.4 nM to 193 nM), or mefloquine (0.5 nM to 254 nM). After 72 hours of incubation, parasitemias were measured by flow cytometry. Cells were stained with 100 nM Mito Tracker Deep Red and 1 × SYBR Green (Thermo Fisher Scientific) in 1 × PBS^84^. IC_50_ values were calculated by non-linear regression analysis.

### DV volume measurements

Dextran-conjugated Oregon Green (OGd) was loaded into parasites as described^18^. Briefly, OGd was dissolved in in 1 × PBS at a final concentration of 25 mg/mL. To 25 μL of the OGd solution we then added 220 μL of a hypotonic solution (5 mM HEPES, 11 mM glucose, and 2 mM MgATP; pH 7.4), pre-warmed to 37 °C. Freshly washed uninfected RBCs (100 μL) were slowly added drop-wise to this mixture. The suspension was gently mixed for 10 minutes until an increase in clarity was observed. 250 μL of pre-warmed hypertonic solution (280 mM NaCl, 40 mM KCl, 11 mM glucose, and 2 mM MgATP; pH 7.4) was then added to reseal the permeabilized OGd-loaded RBCs. The suspension was lightly shaken and centrifuged for 5 minutes at 2,500 rpm. The supernatant was removed, and 10 mL of incomplete medium was added. The cells were gently mixed and centrifuged, and the medium wash was then repeated once more. 10 μL of packed synchronized cells were added to a flask along with 40 μL of packed RBCs loaded with OGd and 5 mL of culture medium. Imaging was started 2 cycles after adding loaded RBCs to 4% parasitemia cultures. To measure the change in DV volume over time, we imaged synchronized parasites every 3 hours for 36 hours starting from merozoite invasion of RBCs (time 0). To measure changes in DV volume with differential amino acid media, iRBCs loaded with OGd were incubated for both 1 hour and 6 hours with AlbuMAXII media containing either isoleucine (Ile) alone or Ile + methionine (Met). After each time point, cells were washed and imaged in media containing Ile only or Ile + Met.

DV images were processed using AutoQuantX2 deconvolution software with the following parameters: spacing: 0.077 × 0.077 × 0.169 (µm); channel: FITC (518 nm); modality: Spinning Disc Confocal; Objective lens: Apo TIRF 100x Oil NA 1.49; Immersion Medium: Oil (1.515). An isosurface was developed using IMARIS software with automatic threshold setting, and DV volumes were calculated for each cell. At least 15 cells were used in each trial to acquire each average DV volume.

### *In vitro* fitness assays

Synchronous mature (late trophozoite/early schizont) Dd2^*Dd2*^ and Dd2^*Dd2 L272F*^ parasites were mixed 1:4 into a single flask and split into two replicate cultures. These cultures were maintained for 40 days (i.e. ~20 generations). DNA was harvested at 48-hour intervals and *pfcrt* was PCR amplified and Sanger sequenced. Allele frequencies were quantified by thymidine versus cytidine intensities corresponding to the *pfcrt* 814 nucleotide in codon 272. A calibration curve was prepared using genomic DNA from each line mixed at various concentrations, to correct for inherent intensity differences between nucleotides as previously described^26^. Allele frequencies were fit using a custom model of the *Plasmodium* intra-erythrocytic life cycle written in R statistical software (version 3.2.3). As described previously^26^, this model fits starting allele frequencies, life cycle stage at first measurement, life cycle lengths, exponential growth rates, and amplification of the genome per generation. An optimal model was obtained by iterative grid searches guided by goodness of fit relative to the empirical data (R^2^).

### Amino acid dependency assay/calculations

Asynchronous Dd2^*Dd2*^ and Dd2^*Dd2 L272F*^ parasite cultures were washed with a variety of media formulations. Our control RPMI media, denoted “RPMI-VLA”, contained all amino acids except for valine, leucine, and alanine, as described^56^. This reconstituted medium was earlier observed to enable parasites to proliferate at the same rate as with RPMI culture medium containing all amino acids^56^. To test for amino acid dependency, Dd2^*Dd2*^ and Dd2^*Dd2 L272F*^ parasites were cultured in amino acid-free RPMI medium supplemented with either 150 μM isoleucine (+I), or 150 μM isoleucine plus: 101 μM methionine (+IM); 101 μM methionine and 200 μM cystine (+IMC); 200 μM cystine (+IC); 200 μM lysine and 200 μM arginine (+IKR); or 150 μM aspartic acid, 136 μM glutamic acid, 200 μM asparagine and 200 μM glutamine (+IDENQ). Every two days for 8 days, an aliquot was taken to measure parasitemia by flow cytometry^56^ while a separate aliquot was subcultured. Growth rates were determined by averaging slopes of linear fits for three independent experiments for Dd2^*Dd2*^ and Dd2^*Dd2 L272F*^ in each type of medium, where the natural log of the product of parasitemia and cumulative dilution factor was plotted against time (*t*) in hours. Data were fitted by linear regression to the equation:

$$\mathrm{ln}(p)=\,\mathrm{ln}(a)+\,\mathrm{ln}(2)\frac{t}{\tau }$$

where *p* = parasitemia × cumulative dilution factor, *a* = starting parasitemia and τ = doubling time. For each medium, mean doubling times were calculated from the three independent experiments.

### Metabolomics analyses

All parasite culturing, metabolite extraction, mass spectrometry, and data acquisition and analysis employed previously established methods^26^. Briefly, double sorbitol synchronized, late-stage (~38 ± 3 hours post-invasion) *P. falciparum* trophozoites were magnetically purified using CS columns mounted on a SuperMACS magnetic separator (Miltneyi Biotec). Eluted iRBCs were resuspended at 0.4% hematocrit and incubated for 2 hours at 37 °C. Following centrifugation, iRBC pellets were resuspended in cold (4 °C) 90% methanol, homogenized by vortexing, and centrifuged at 10,000 × g for 5 minutes at 4 °C. Supernatants were harvested and were stored at −80 °C prior to use. Before metabolomics analysis, samples were dried under a stream of N_2_ and were resuspended at a 4:1 dilution (relative to the original iRBC pellet volume) in HPLC-grade water. High-resolution mass spectrometry data were acquired on a Thermo Fisher Exactive Mass spectrometer in negative mode using 25-minute reverse phase gradients and ion-pairing chromatography^85^. Metabolites were identified relative to the known retention times of standards and metabolite signals were quantified using MAVEN^86^. The absolute concentration of each peptide was quantified using calibration curves constructed from synthetic peptide standards, as described^26^. All data analyses and statistical test were conducted using custom in-house software written in the R statistical software environment.

### Measurement of intracellular calcium

Doubly sorbitol-synchronized trophozoite stage parasites, harvested 30–34 hours post-invasion, were isolated from iRBCs with 0.05% saponin in 1 × PBS. Liberated parasites were centrifuged at 8,000 rpm for 10 minutes at 4 °C to remove RBC membranes. Parasite pellets were washed with Buffer A (116 mM NaCl, 5.4 mM KCl, 0.8 mM MgSO_4_, 5.5 mM D-Glucose, 50 mM HEPES, 2 mM CaCl_2_ and 40 μM probenecid; pH 7.2) and incubated in same buffer for 1 hour at 37 °C with the fluorescent, free Ca^2+^ indicator Fluo-4 AM (5 μM in DMSO; Invitrogen). Probenecid was included as an inhibitor of organic anion transport^72^. Cells were then washed three times in Buffer A to remove extracellular stain and resuspended in Buffer A only. Fluorescence for each sample was then measured every second for 500 seconds using an F-4500 Hitachi spectrofluorometer with excitation at 488 nm and emission at 530 nm at 37 °C. At t = 50 to 60 seconds, each parasite line was treated with 25 μM Monensin, 10 μM Nigericin, or 80 μM CQ. Control samples were treated with in *N*,*N*-Dimethylformamide (DMF), DMSO, and Buffer A, respectively. Free Ca^2+^ concentration was calculated using the F-4500 Intracellular Cation Measurement System, version 1.02 (Hitachi), where:

$${[{{\rm{Ca}}}^{2+}]}_{c}={{\rm{k}}}_{d}(\frac{F\,-{{\rm{F}}}_{min}}{{{\rm{F}}}_{max}-{\rm{F}}})$$

k_*d*_ = 345 nM, F = measured fluorescence intensity, F_*max*_ = fluorescence in the presence of digitonin, and F_*min*_ = fluorescence in the presence of digitonin and 15 mM EGTA. Results are expressed as mean ± SEM of nine independent experiments. Statistical differences were assessed using Mann-Whitney *U* tests.

## Electronic supplementary material

Supporting Information

## Data Availability

All data generated or analyzed during this study are included in the main text or supplement of this published article.
