# Supplementary material for: Evidence for Regulation of Hemoglobin Metabolism and Intracellular Ionic Flux by the Plasmodium falciparum Chloroquine Resistance Transporter
Source: Sci Rep. 2018 Sep 11;8:13578. doi: 10.1038/s41598-018-31715-9 (PMC6134138; doi:10.1038/s41598-018-31715-9)
Supplement: Supplementary file 1 — Supporting Information [file 41598_2018_31715_MOESM1_ESM.pdf]

SUPPORTING INFORMATION

Evidence for Regulation of Hemoglobin Metabolism and Intracellular Ionic Flux by the *Plasmodium falciparum* Chloroquine Resistance Transporter

Andrew H. Lee, Satish K. Dhingra, Ian A. Lewis, Maneesh K. Singh, Amila Siriwardana, Seema Dalal, Kelly Rubiano, Matthias S. Klein, Katelynn S. Baska, Sanjeev Krishna, Michael Klemba, Paul D. Roepe, Manuel Llinás, Celia R.S. Garcia, David A. Fidock

TABLE OF CONTENTS

Supplementary Table S1. Mean ± SEM IC_{50} values (nM) of *pfcrt*-modified lines.

Supplementary Table S2. Mean±SEM digestive vacuole volumes of *pfcrt*-modified lines.

Fig. S1. The PfCRT L272F mutation in the large vacuolar loop creates an enlarged digestive vacuole phenotype.

Fig. S2. Hemoglobin peptide profiles of the L272F mutant and isogenic Dd2 control lines.

Fig. S3. Metabolic profiling of the L272F mutant reveals altered peptide accumulation.

Fig. S4. PfCRT is not a contributing factor in Ca^{2+} release from the endoplasmic reticulum.

Supplementary Table S1. Mean±SEM IC_{50} values (nM) of *pfcrt*-modified lines.

Line	Dd2	$Dd2^{Dd2}$	$Dd2^{Dd2\ L272F}$	GC03
**CQ IC_{50} (nM)**	**91.3 ± 5.5**	**78.1 ± 7.4**	**17.6 ± 1.9**	**10.1 ± 2.3**
# assays	5	5	5	5
p value vs $Dd2^{Dd2}$	0.31		0.008	0.008
p value vs GC03	0.008		0.10	
**md-CQ IC_{50} (nM)**	**573.4 ± 53.1**	**512.8 ± 48.4**	**106.8 ± 10.1**	**19.3 ± 3.3**
# assays	6	6	6	6
p value vs $Dd2^{Dd2}$	0.39		0.002	0.002
p value vs GC03	0.002		0.002	
**md-ADQ IC_{50} (nM)**	**33.0 ± 5.8**	**25.8 ± 4.4**	**15.6 ± 3.4**	**12.1 ± 2.9**
# assays	6	6	6	6
p value vs $Dd2^{Dd2}$	0.31		0.14	0.09
p value vs GC03	0.002		0.49	
**CQ + VP IC_{50} (nM)**	**19.7 ± 0.7**	**19.5 ± 2.1**	**9.4 ± 1.3**	**13.2 ± 0.8**
# assays	4	4	4	4
p value vs $Dd2^{Dd2}$	0.69		0.03	0.06
p value vs GC03	0.03		0.11	
**md-CQ + VP IC_{50} (nM)**	**101.6 ± 10.8**	**99.4 ± 8.0**	**23.1 ± 5.0**	**24.0 ± 2.2**
# assays	4	4	4	4
p value vs $Dd2^{Dd2}$	>0.99		0.03	0.03
p value vs GC03	0.03		>0.99	
**md-ADQ + VP IC_{50} (nM)**	**13.4 ± 2.4**	**13.3 ± 3.0**	**11.3 ± 1.3**	**11.9 ± 2.8**
# assays	5	5	5	5
p value vs $Dd2^{Dd2}$	>0.99		0.55	0.69
p value vs GC03	0.60		0.84	
**LMF IC_{50} (nM)**	**3.7 ± 0.7**	**4.0 ± 0.8**	**4.1 ± 1.0**	**4.7 ± 1.4**
# assays	3	3	3	3
p value vs $Dd2^{Dd2}$	>0.99		>0.99	0.70
p value vs GC03	>0.99		>0.99	
**MFQ IC_{50} (nM)**	**22.1 ± 1.7**	**26.7 ± 2.4**	**29.9 ± 2.7**	**14.7 ± 2.2**
# assays	4	4	4	4
p value vs $Dd2^{Dd2}$	0.20		0.34	0.03
p value vs GC03	0.06		0.03	
**PND IC_{50} (nM)**	**5.1 ± 0.5**	**5.6 ± 0.7**	**7.4 ± 0.4**	**8.6 ± 1.1**
# assays	3	3	3	3
p value vs $Dd2^{Dd2}$	0.40		0.10	0.10
p value vs GC03	0.10		0.40	

Mean±SEM IC_{50} values are represented in nM. IC_{50} vaues were determined from 3–6 independent assays performed in duplicate. CQ, chloroquine; md-CQ, monodesethyl-chloroquine; md-ADQ, monodesethyl-amodiaquine; VP, verapamil; LMF, lumefantrine; MFQ, mefloquine; PND, pyronaridine. Statistical comparisons of each line to the recombinant $Dd2^{Dd2}$ control were made using non-parametric Mann-Whitney *U* tests.

Shading code: ns | *$p<0.05$ | **$p<0.01$

Supplementary Table S2. Mean±SEM digestive vacuole volumes of *pfcrt*-modified lines.

Line	$Dd2^{Dd2}$	$Dd2^{Dd2\ L272F}$	$Dd2^{GC03}$	Fold Change/$Dd2^{Dd2}$	
				$Dd2^{Dd2\ L272F}$	$Dd2^{GC03}$
**t=15 hours**	**0.96 ± 0.14**	**1.22 ± 0.08**	**0.80 ± 0.08**	**1.27**	**0.83**
# assays	20	20	20		
p value vs $Dd2^{Dd2}$		0.07	0.51		
p value vs $Dd2^{GC03}$		0.0003			
**t=18 hours**	**1.38 ± 0.22**	**2.55 ± 0.17**	**1.07 ± 0.08**	**1.85**	**0.78**
# assays	20	20	20		
p value vs $Dd2^{Dd2}$		<0.0001	0.70		
p value vs $Dd2^{GC03}$		<0.0001			
**t=21 hours**	**1.98 ± 0.19**	**3.45 ± 0.23**	**1.70 ± 0.14**	**1.74**	**0.86**
# assays	20	20	20		
p value vs $Dd2^{Dd2}$		<0.0001	0.55		
p value vs $Dd2^{GC03}$		<0.0001			
**t=24 hours**	**2.99 ± 0.25**	**4.59 ± 0.25**	**1.95 ± 0.24**	**1.54**	**0.65**
# assays	20	20	20		
p value vs $Dd2^{Dd2}$		0.0002	0.008		
p value vs $Dd2^{GC03}$		<0.0001			
**t=27 hours**	**4.00 ± 0.33**	**5.78 ± 0.62**	**2.10 ± 0.18**	**1.45**	**0.53**
# assays	20	20	20		
p value vs $Dd2^{Dd2}$		0.04	<0.0001		
p value vs $Dd2^{GC03}$		<0.0001			
**t=30 hours**	**5.36 ± 0.37**	**6.33 ± 0.44**	**2.19 ± 0.19**	**1.18**	**0.41**
# assays	20	20	20		
p value vs $Dd2^{Dd2}$		0.09	<0.0001		
p value vs $Dd2^{GC03}$		<0.0001			
**t=33 hours**	**5.89 ± 0.51**	**7.09 ± 0.59**	**2.36 ± 0.28**	**1.20**	**0.40**
# assays	20	20	20		
p value vs $Dd2^{Dd2}$		0.13	<0.0001		
p value vs $Dd2^{GC03}$		<0.0001			

Mean±SEM digestive vacuole volumes represented in μm^3/cell. Volumetric measurements were carried out in 20 independent experiements. Statistical comparisons of each line to the recombinant $Dd2^{Dd2}$ control were made using non-parametric Mann-Whitney *U* tests.

Shading code:	ns	**p*<0.05	***p*<0.01	****p*<0.001

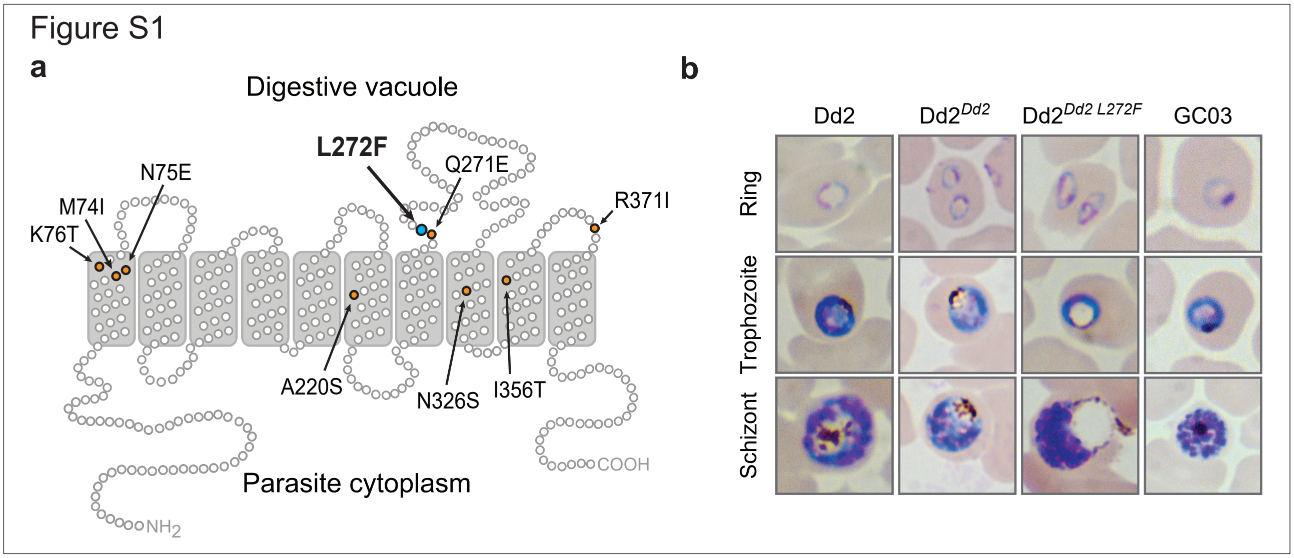

Figure S1. The PfCRT L272F mutation in the large vacuolar loop creates an enlarged digestive vacuole phenotype. (**a**) Schematic of PfCRT predicted topology. The L272F mutation (blue dot) is predicted to localize to a large loop, extending into the DV, between transmembrane helices 7 and 8. The other mutations listed are present in the Dd2 mutant protein that mediates CQ resistance and that differs from the wild-type sequence at 8 positions (orange dots: M74I, N75E, K76T, A220S, Q271E, N326S, I356T, and R371I). (**b**) Representative images of Dd2, Dd2^{Dd2}, Dd2$^{Dd2\ L272F}$, and GC03 parasites at ring, trophozoite, and schizont stages. Parasites were Giemsa-stained and visualized by light microscopy. Dd2$^{Dd2\ L272F}$ exhibited enlarged, translucent DVs in trophozoite and schizont stages when Hb catabolism is the most active.

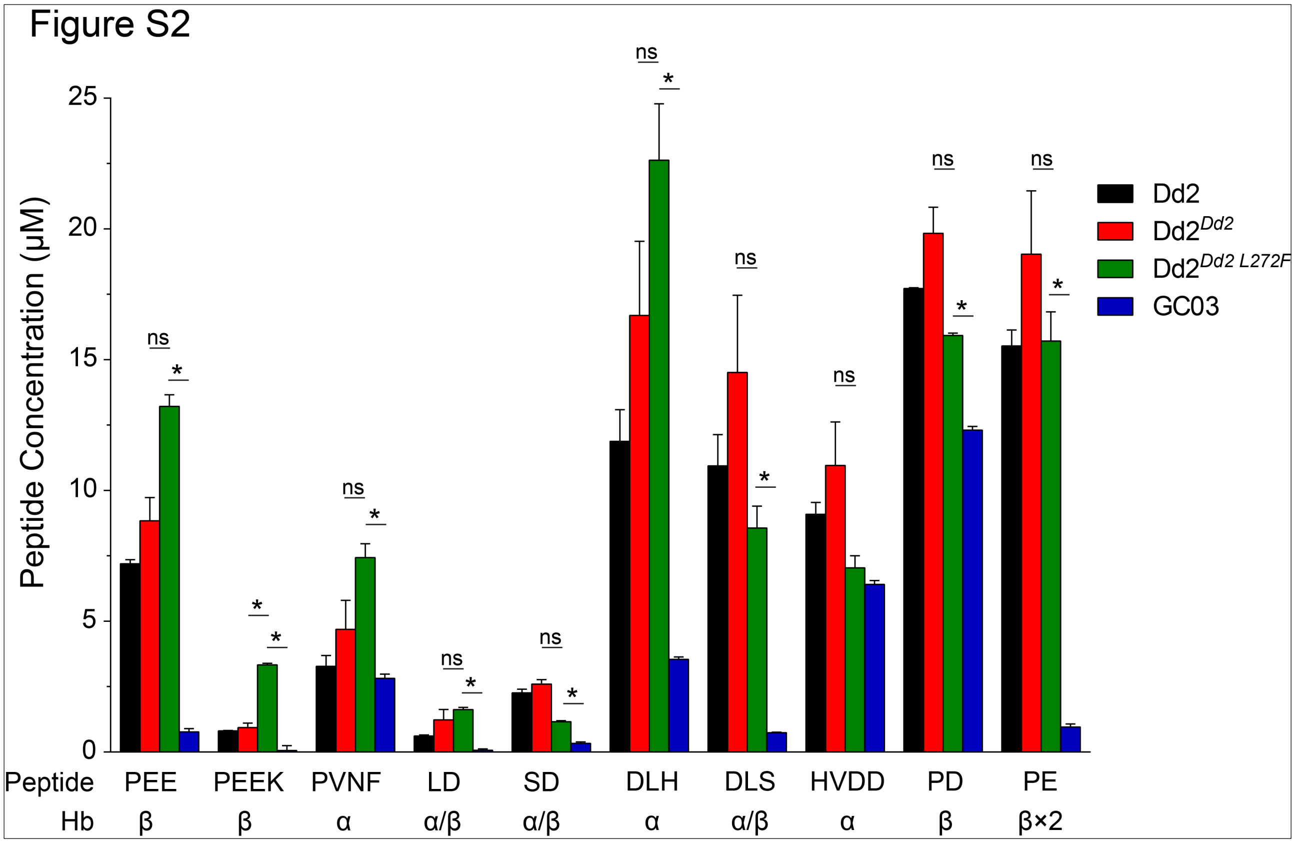

Figure S2. Hemoglobin peptide profiles of the L272F mutant and isogenic Dd2 control lines. Concentrations per parasite of short, Hb-derived peptides for Dd2 (black), Dd2^{Dd2} (red), Dd2$^{Dd2\ L272F}$ (green), and the CQS strain GC03 (blue) as measured by LC-MS for three independent assays. Data were obtained from three independent experiments and are presented as means±SEM. Two-tailed Student *t* tests were performed to compare Dd2$^{Dd2\ L272F}$ versus either Dd2^{Dd2} or GC03. ns, not significant; *$p<0.05$.

Figure S3 (next page). Metabolic profiling of the L272F mutant reveals altered peptide accumulation. Standard scores (z-scores) of metabolites measured by LC-MS for Dd2 (black), Dd2^{Dd2} (red), and Dd2$^{Dd2\ L272F}$ (green). Each bar represents the number of standard deviations for each metabolite such that $z = (x - \mu)/\sigma$, where x = signal of a metabolite (e.g. PEEK), μ = mean signal of a metabolite for 3 independent assays, and σ = the standard deviation for the same metabolite in Dd2.

Figure S3

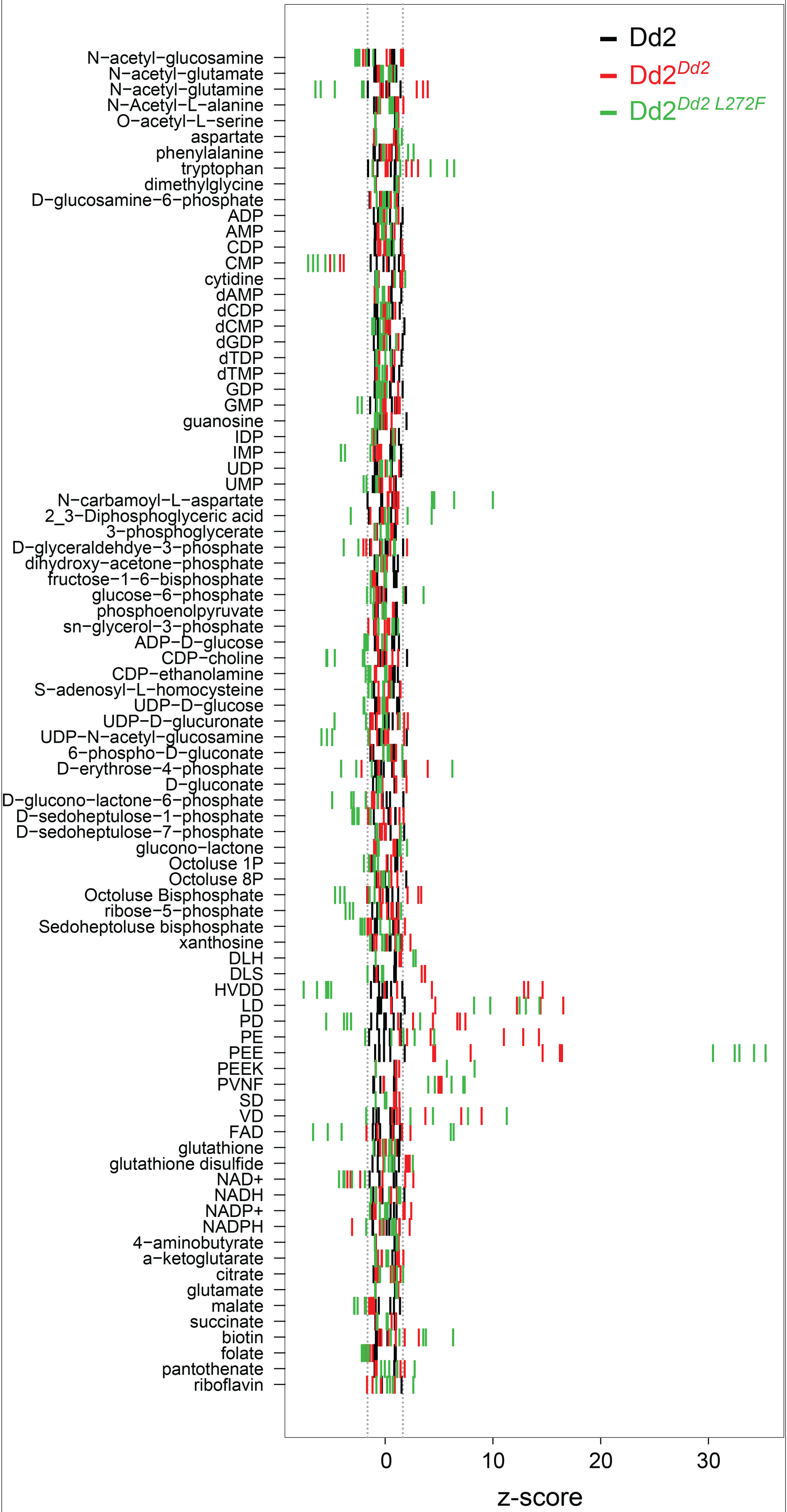

Dd2
Dd2^Dd2
Dd2^Dd2 L272F
N−acetyl−glucosamine
N−acetyl−glutamate
N−acetyl−glutamine
N−Acetyl−L−alanine
O−acetyl−L−serine
aspartate
phenylalanine
tryptophan
dimethylglycine
D−glucosamine−6−phosphate
ADP
AMP
CDP
CMP
cytidine
dAMP
dCDP
dCMP
dGDP
dTDP
dTMP
GDP
GMP
guanosine
IDP
IMP
UDP
UMP
N−carbamoyl−L−aspartate
2_3−Diphosphoglyceric acid
3−phosphoglycerate
D−glyceraldehdye−3−phosphate
dihydroxy−acetone−phosphate
fructose−1−6−bisphosphate
glucose−6−phosphate
phosphoenolpyruvate
sn−glycerol−3−phosphate
ADP−D−glucose
CDP−choline
CDP−ethanolamine
S−adenosyl−L−homocysteine
UDP−D−glucose
UDP−D−glucuronate
UDP−N−acetyl−glucosamine
6−phospho−D−gluconate
D−erythrose−4−phosphate
D−gluconate
D−glucono−lactone−6−phosphate
D−sedoheptulose−1−phosphate
D−sedoheptulose−7−phosphate
glucono−lactone
Octoluse 1P
Octoluse 8P
Octoluse Bisphosphate
ribose−5−phosphate
Sedoheptoluse bisphosphate
xanthosine
DLH
DLS
HVDD
LD
PD
PE
PEE
PEEK
PVNF
SD
VD
FAD
glutathione
glutathione disulfide
NAD+
NADH
NADP+
NADPH
4−aminobutyrate
a−ketoglutarate
citrate
glutamate
malate
succinate
biotin
folate
pantothenate
riboflavin
0
10
20
30
z-score

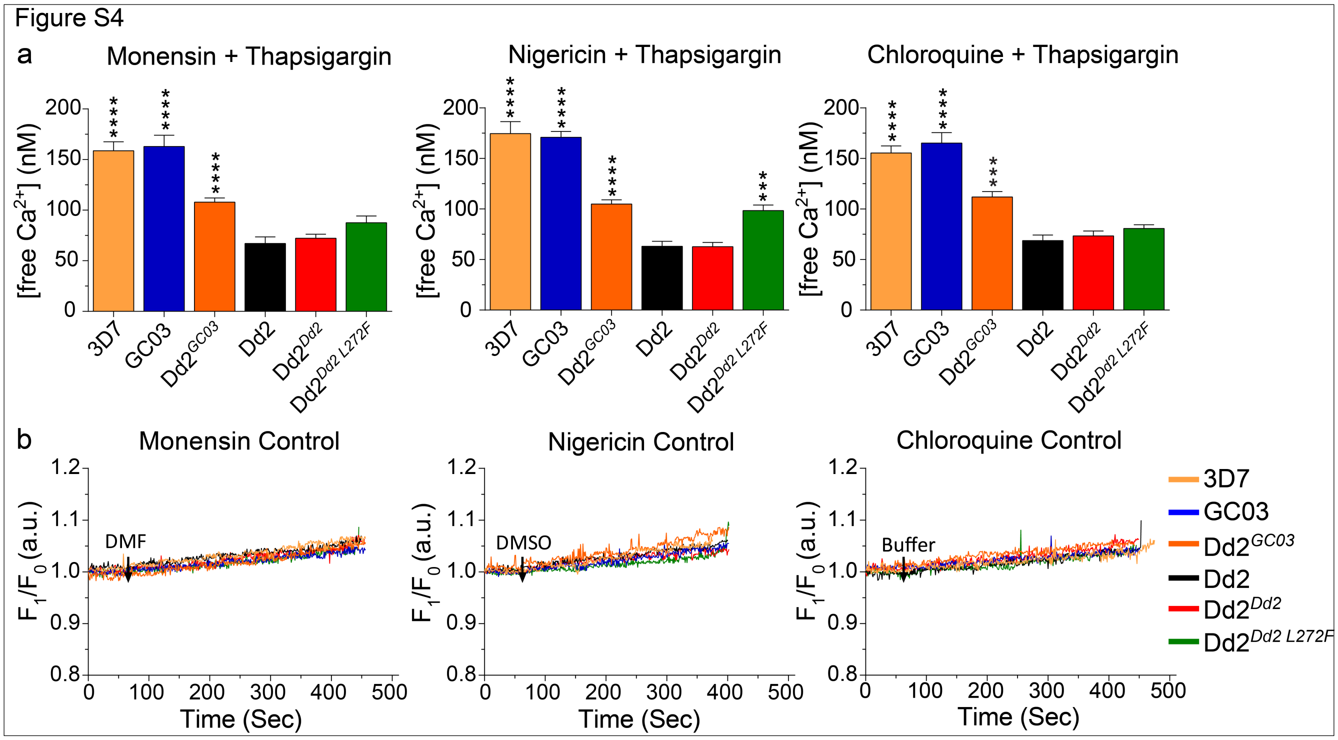

Figure S4. PfCRT is not a contributing factor in Ca^{2+} release from the endoplasmic reticulum. Efflux of free Ca^{2+} into the cytoplasm treated with ionophores was measured using the fluorescent dye Fluo-4 AM for the strains 3D7 (light orange), GC03 (blue), Dd2^{GC03} (dark orange), Dd2 (black), Dd2^{Dd2} (red), and Dd2$^{Dd2\ L272F}$ (green). Each strain was independently treated with monensin (25 μM) plus thapsigargin, nigericin (10 μM) plus thapsigargin, or chloroquine (80 μM) plus thapsigargin. Thapsigargin was used at 5 μM. Results are shown as means ± SEM, derived from nine independent experiments. ***$p< 0.001$, ****$p< 0.0001$; two-tailed Mann-Whitney *U* test relative to Dd2^{Dd2}. **(b)** Traces observed with mock (vehicle)-treated parasites. DMF, Dimethylformamide (solvent for monensin); DMSO, Dimethyl sulfoxide (solvent for nigericin); buffer is water (solvent for chloroquine).
